# Black gold rush - Evaluating the efficiency of the Fractionator in separating Hymenoptera families in a meadow ecosystem over a two week period

**DOI:** 10.3897/BDJ.11.e107051

**Published:** 2023-10-23

**Authors:** Maura Haas-Renninger, Noa L. A. Schwabe, Marina Moser, Lars Krogmann

**Affiliations:** 1 State Museum of Natural History Stuttgart, Stuttgart, Germany State Museum of Natural History Stuttgart Stuttgart Germany; 2 University of Hohenheim, Stuttgart, Germany University of Hohenheim Stuttgart Germany

**Keywords:** bulk samples, biomass, monitoring, insect decline, taxonomic impediment

## Abstract

In the face of insect decline, monitoring projects are launched widely to assess trends of insect populations. Collecting over long time periods results in large numbers of samples with thousands of individuals that are often just stored in freezers waiting to be further processed. As the time-consuming process of sorting and identifying specimens prevents taxonomists from working on mass samples, important information on species composition remains unknown and taxonomically neglected species remain undiagnosed. Size fractioning of bulk samples can improve sample handling and, thus, can help to overcome the taxonomic impediment. In this paper, we evaluate the efficiency of the fractionator in separating Hymenoptera families from a Malaise trap sample of a meadow ecosystem over a two week interval to make them available for further morphological identification. The fractionator system by Buffington and Gates (2008) was used to separate the sample in two size classes – a large (macro) and a small (micro) fraction – and Hymenoptera specimens were then counted and identified on family level. In total, 2,449 Hymenoptera specimens were found in the macro fraction and 3,016 in the micro fraction (5,465 specimens in total). For 24 out of 34 Hymenoptera families (71%), separation was significant. This study illustrates the efficiency of the fractionator and its potential to improve workflows dealing with specimen-rich Malaise trap samples.

## Introduction

In times of global insect decline (Maes and van Dyck 2001, Brooks et al. 2012, Hallmann et al. 2017, Lister and Garcia 2018), insect diversity assessments and monitoring schemes become more important than ever. In the past decades, many insect monitoring projects were launched (e.g. Seibold et al. (2019), Karlsson et al. (2020), Lehmann et al. (2021), Staab et al. (2023)). Malaise traps are widely used to assess population trends of flying insects, as they are standardised traps that are very efficient in collecting flying insects (Matthews and Matthews 1983, Ssymank et al. 2018, Karlsson et al. 2020). Malaise trap samples comprise large numbers of insects from different orders and with high specimen size heterogeneity (Geiger et al. 2016). The largest amount is made up of just two orders, Hymenoptera and Diptera (Srivathsan et al. 2023), which are the most species-rich insect orders (Zhang 2011, Forbes et al. 2018). These groups contain a significant number of so-called ‘dark taxa’, small-sized specimens that lack experts to identify them (Hausmann et al. 2020, Chimeno et al. 2022), which contributes to the taxonomic impediment (Engel et al. 2021). These samples are highly valuable as they are likely to contain undescribed species. Sorting and identifying specimens from large bulk samples is time-consuming and constitutes the main bottleneck of a morphological approach (Piper et al. 2019). Due to the lack of sorting, most of the collected material is stored indefinitely in freezers, awaiting proper identification. Implementation of a strategy for downstream sample handling after biomass assessment heavily depends on the study goal. However, with regards to millions of unidentified specimens, the high potential and value of these samples provide sufficient reason to develop a comprehensive concept for each monitoring approach, as an accurate identification of a species forms the base of every effort made in the fields of ecology, biogeography and conservation.

Metabarcoding is a highly promising method for molecular identification of species from mass samples (Taberlet et al. 2012). In this approach, DNA is extracted from the whole bulk sample (Hajibabaei et al. 2011). A standardised marker region of the mitochondrial cytochrome c oxidase I gene (COI) is amplified and after high-throughput sequencing (Shendure and Ji 2008), the sequences are compared against a reference database for species identification (Ratnasingham and Hebert 2007, Hajibabaei et al. 2011). For metabarcoding, no taxonomic knowledge of experts is needed and it is, therefore, a cost-efficient approach for biodiversity assessments (Hajibabaei et al. 2011, Elbrecht et al. 2017b). However, the molecular identification of a species is limited by the availability of an accurately identified reference barcode (Ekrem et al. 2007, Virgilio et al. 2010). Concerning dark taxa, the lack of reference data results in large numbers of OTUs (operational taxonomic units), whose exact number depends largely on ambiguous species cut-off levels and which cannot be linked to a species name. Without this linkage, we are unable to grasp the biology of a species, its ecological requirements and, therefore, miss the initial goal to learn about species in order to protect them. In addition, small-sized taxa remain undetected, as they contribute less DNA compared to larger specimens (Elbrecht et al. 2017a). Additionally, homogenisation of bulk samples to extract DNA from the entire “biodiversity soup” precludes the possibility to study morphological characteristics of individual specimens and would ultimately not allow for voucher specimens to be made accessible to taxonomic research through their preservation in collections.

The primary impediment of a conventional monitoring approach is the vast number of insect specimens that need to be identified and preserved for extended periods. In this context, Buffington and Gates (2008) presented a cost- and time-efficient method for size-sorting of specimens from large bulk samples: the fractionator. In this approach, the major aim was not genetic identification using size fractions, but rather recovering intact specimens from mass samples for subsequent morphological analysis. The fractionator is a system based on a sieve in a plastic tub that is placed on an orbital shaker. Full fluid-conserved insect samples are immersed in soapy water to reduce surface tension. Stacking the tubs enables the fractionation of multiple samples simultaneously. As a result, specimens from the bulk samples are sorted into two size classes: a large (macro) and a small (micro) fraction. In a recent paper, Elbrecht et al. (2021) showed that size fractioning of specimens from insect bulk samples increases taxon recovery in a metabarcoding concept. However, to our knowledge, the fractionator method is still not widely utilised. In this paper, we evaluate the efficiency of the fractionator to separate Hymenoptera families from a two week Malaise trap sample of a meadow ecosystem to make them available for morphological identification by testing which Hymenoptera families are found to what extent in the different size fractions.

## Methods

### Sample collection

For our study, we used a sample from the Malaise trap project “Aerial Biomass”, which is a subproject of the insect monitoring project in south-western Germany that was launched in 2018 by the State Institute for Environment Baden-Wuerttemberg (LUBW). This project aims to evaluate the biomass of flying insects throughout the year in various nature conservation, grassland and agricultural areas. The traps and protocols used are standardised and based on the recommendations by the Entomological Society Krefeld (Ssymank et al. 2018). We used a Malaise trap sample from the nature conservation area “Apfelberg” near Karlsruhe, Baden-Württemberg (Germany, 49.16754°N; 8.7903°E (DD)). The site is an extensively managed meadow with southern exposure so that the trap was fully sun-exposed. The sampling area is surrounded by a dense growth of woody plants, including fruit trees and bushes, towards the north and west. Vineyards and agricultural fields are located beyond this structure. Towards the east, the area is richly structured with woodlands and small patches of grassland, followed by farmland. The site slopes towards the south, which is dominated by meadows and agricultural sites. The plant community within a radius of 50 m surrounding the trap can be described as an oat grass meadow with elements of a semi-arid grassland. The Malaise trap was provided and placed in the site by members of the Entomological Society of Krefeld. This trap is a bi-coloured model, based on the Malaise trap model by Henry Townes (Townes 1972, Matthews and Matthews 1983). Our sample contained arthropods from a two-week sampling interval in summer between 18 June and 3 July 2019. The sampling bottle contained 80% ethanol denatured with 1% MEK. Ethanol concentration was checked when the bottle was collected after two weeks and the ethanol concentration was re-adjusted to 80%. The sample was stored in a freezer at -20°C at Stuttgart State Museum of Natural History (SMNS) until further processing.

### Fractionation

The fractionator setup and protocol are based on Buffington and Gates (2008). We used a vegetable strainer with a mesh size of 2 mm (“Ikea” Idealisk 501.037.55, Delft, Netherlands) which fits into a plastic tub (“Ikea” Samla 694.408.36, Delft, Netherlands). After the bulk sample was poured in the sieve, 3-3.5 l of tap water with three drops of dish soap was added to the sample to reduce surface tension. The orbital shaker (“IKA Labortechnik” KS 501 digital, Germany) was set to 38-40 motions per minute and the sample was fractionated for 30 min. The duration of the fractioning was set by experience (Schweizer, personal communication) as this was long enough to separate large from small specimens before they could start to disintegrate as a result of overlong exposure to the detergent. Afterwards, the micro fraction was obtained from the plastic tub with the soapy water using a round metal tea sieve (mesh size 0.25 mm) and soapy water was discarded. The micro fraction was transferred to a 50 ml FalconTM tube with fresh 80% denatured ethanol (+ 1% MEK). The macro fraction was obtained by inverting the strainer over the empty plastic tub and carefully beating the strainer so that the specimens fell into the tub. Remaining specimens were removed with 80% denatured ethanol from a squeeze bottle and transferred to a separate plastic container for storage. The fractionating process, including the transfer to vials with this adapted protocol, takes 45 minutes per bulk sample. As two tubs can be placed above each other during fractioning, two samples can be fractioned at the same time with a total duration of 1 hour including handling time after fractionation.

### Identification and data analysis

All Apocrita specimens from both fractions were sorted out, counted and identified to family level using Goulet and Huber (1993). Specimens of the suborder ‘Symphyta’ were not further identified. As the sample we used for this study was obtained from the insect monitoring project, all wild bees (Anthophila) were manually separated before fractioning and were consequently excluded from our study. The specimens were stored in 99.6% pure ethanol with one vial per family and fraction for further analysis. Separation of specimens was considered efficient, when ≥ 95% of specimens were found in one of the two size fractions.

## Results

**Figure 1.**
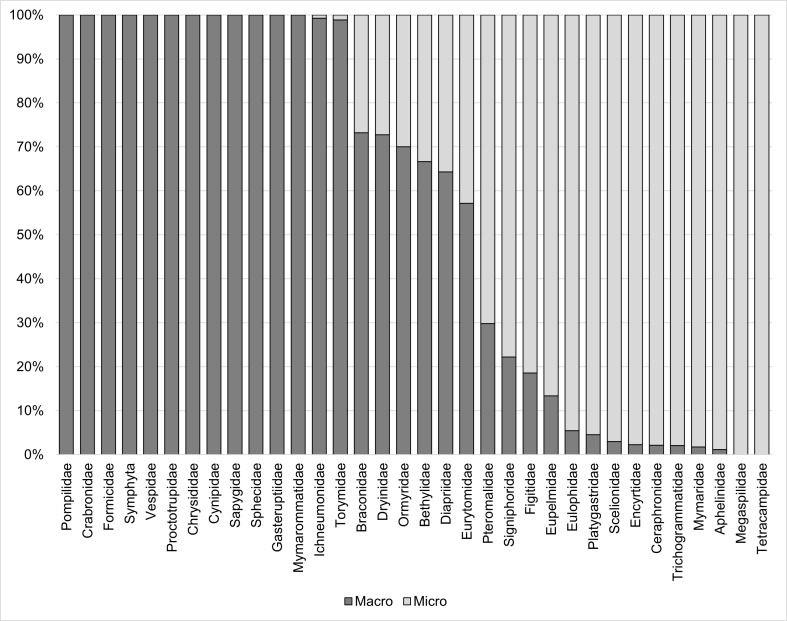
Efficiency of size fractionation for Hymenoptera families (including ‘Symphyta’). For specimen numbers, see Table 1.

Overall, 2,449 Hymenoptera specimens were included in the macro fraction and 3,016 in the micro fraction (5,465 specimens in total, Table 1). Specimens were intact, clean and free of Lepidoptera scales after fractionation. All Apocrita were identified to family level, which resulted in a total of 33 families. For 24 out of 34 families (including ‘Symphyta’), separation was efficient, corresponding to 71% of all sorted groups. The families Crabronidae, Sphecidae, Torymidae, Chrysididae, Cynipidae, Gasteruptiidae, Ichneumonidae, Mymarommatidae, Proctotrupidae, Formicidae, Pompilidae, Sapygidae, Vespidae and ‘Symphyta’ were efficiently separated into the macro fraction (Fig. 1, Table 1). For the micro fraction, the families Ceraphronidae, Megaspilidae, Aphelinidae, Encyrtidae, Eulophidae, Mymaridae, Tetracampidae, Trichogrammatidae, Platygastridae and Scelionidae were separated efficiently. Separation was not efficient for the families Eupelmidae, Eurytomidae, Ormyridae, Pteromalidae, Signiphoridae, Bethylidae, Dryinidae, Figitidae, Diapriidae and Braconidae.

## Discussion

This study illustrates the efficiency of the fractionator by Buffington and Gates (2008) for Hymenoptera specimens in a meadow ecosystem over a two-week period and its potential to improve workflows dealing with specimen-rich Malaise trap samples. The results show that 71% of Hymenoptera families including the suborder ‘Symphyta’ were separated efficiently for this sample. For the families Eupelmidae, Eurytomidae, Ormyridae, Pteromalidae, Signiphoridae, Bethylidae, Dryinidae, Figitidae, Diapriidae and Braconidae, separation was not clear. This is due to the small number of total specimens for some of these families (e.g. Signiphoridae) and the size range of specimens that varies within different taxa of these groups (e.g. Braconidae). The best way to work with such families is to know the approximate size range of a specific subfamily or species group and choose the fraction accordingly. In some cases, it would be necessary to have a look at both size fractions. The single Mymarommatidae specimen in this sample ended up in the macro fraction. Species of the family Mymarommatidae have a maximum size of 0.75 mm (Noyes 2019). The single specimen found in this sample, therefore, most likely ended up in the macro fraction by chance. One limitation of our study is that it is based on a single Malaise trap sample from a grassland site in a two week interval. Therefore, the size distribution of taxa of the same family might differ considering their phenology, which means that our results may look different at another point in time of the year. Given the enormous number of specimens and the large diversity of hymenopteran families that we found, we are, nevertheless, confident that our dataset represents a good proxy for the efficiency of the fractionator.

Buffington and Gates (2008) chose a fractioning duration of 2 hours. Our results show that most families of Hymenoptera can be separated effectively with a fractioning duration of 30 minutes. This is a quarter of the duration used by Buffington and Gates (2008) and, therefore, saves a considerable amount of time. Further, specimens spend less time in the soapy water with low concentrations of ethanol and can be transferred faster to high percentage ethanol for better conservation. To have the option for subsequent analyses, it is advisable to preserve 50 ml of the initial ethanol in a vial before the sample is poured into the sieve for fractionation. In a recent paper, Brühl et al. (2021) used ethanol from Malaise trap samples to detect pesticide residues in nature conservation areas. This shows that not only the insect specimens, but also the ethanol in which the insects were conserved, have high potential to answer questions in the field of nature conservation.

The benefits of using the fractionator for sorting out microhymenoptera were already highlighted in Buffington and Gates (2008) (e.g. clean specimens) and we can add that it facilitates the sorting process as microhymenoptera tend to get stuck in hairs and bristles of larger Hymenoptera and Diptera specimens. Here, we want to highlight the advantages of including the fractionator in a standard Malaise trap monitoring workflow. The proposed method makes large bulk samples more easily accessible and attractive to work with, as experts do not need to spend time manually sorting through massive amounts of material. It can easily be implemented in the monitoring workflow, as it is time- and cost-efficient. To minimise cross-contamination, particularly during subsequent molecular analysis, it is recommended to sterilise the fractionation equipment between samples. Further, Elbrecht et al. (2017a) showed that sample presorting reduces sequencing depth five-fold in a metabarcoding approach resulting in reduced sequencing costs. They also showed that 30% more taxa can be recovered in sorted compared to unsorted samples. Size fractionation is also used in the workflow of the “DiversityScanner”, a very recent approach in which insect specimens from bulk samples are identified and processed by a machine (Wührl et al. 2022). However, fractioning samples also comes with some costs, such as doubling of sample containers and labels, which need to be kept track of. Some specimens might get damaged, as, for example, antenna of Ichneumonoidea and Diptera, especially in Nematocera. Therefore, benefits and costs of the fractioning method need to be considered before implementing it into the workflow of mass sample handling.

Malaise traps are ideal to catch Hymenoptera and Diptera (Geiger et al. 2016). Globally, 16 out of the 20 most commonly found insect families can be found in Malaise trap samples (Srivathsan et al. 2023), of which six Hymenoptera families are also found in this study. These samples are, therefore, a ‘bonanza’ for the diversity of these dark taxa (Zhang 2011, Forbes et al. 2018). Most Malaise trap monitoring approaches focus solely on insect biomass as it is cheap and easy to implement (Ssymank et al. 2018). Elbrecht et al. (2021) described the restrictions of metabarcoding approaches as small taxa often remain undetected. Further, samples are mechanically homogenised and end up as “biodiversity soup”, unable to be identified morphologically later on. Depending on the study goal, metabarcoding might be the choice for qualitative analysis of diversity as discussed in Elbrecht et al. (2017a). For quantitative assessments and especially for taxonomic approaches, it is, however, essential to retain intact specimens in order to preserve their morphological diversity.

In the light of biodiversity loss, collections of natural history museums are highly relevant as they can function as windows to the past and contribute significant data for the documentation of species declines (Shaffer et al. 1998). Scientists at natural history museums publish in the fields of systematics, evolution and nature conservation. As taxonomic experts are rare and descriptions and identifications of some species groups are inadequate, proper taxonomic work, including describing species and preserving voucher specimens in collections, is more important than ever. In this context, insect mass samples from monitoring projects are of high potential because they often contain new species even in supposedly well-studied regions (Moser et al. 2023).

We hope that our results provide an incentive for Hymenopterists to further investigate the morphological diversity associated with the study of the ‘black gold’. Automating parts of the sorting process, allows taxonomists to allocate their time towards much-needed taxonomic research. As a result, the fractionator has the potential to help overcome the taxonomic impediment.

## Figures and Tables

**Table 1.** Number of Hymenoptera specimens per family (including ‘Symphyta’) and size fraction. n: number of specimens, Eff: efficiency: percentage of specimens in the fraction with the larger amount of specimens, fraction: fraction with the larger amount of specimens

Superfamily	Family	Macro, n = 2,449	Micro, n = 3,016	Total, n = 5,465	Eff	Fraction
Apoidea	Crabronidae	118	0	118	100%	macro
Apoidea	Sphecidae	2	0	2	100%	macro
Ceraphronoidea	Ceraphronidae	2	92	94	98%	micro
Ceraphronoidea	Megaspilidae	0	13	13	100%	micro
Chalcidoidea	Aphelinidae	1	86	87	99%	micro
Chalcidoidea	Encyrtidae	13	566	579	98%	micro
Chalcidoidea	Eulophidae	27	472	499	95%	micro
Chalcidoidea	Eupelmidae	2	13	15	87%	micro
Chalcidoidea	Eurytomidae	12	9	21	57%	macro
Chalcidoidea	Mymaridae	9	506	515	98%	micro
Chalcidoidea	Ormyridae	7	3	10	70%	macro
Chalcidoidea	Pteromalidae	56	132	188	70%	micro
Chalcidoidea	Signiphoridae	4	14	18	78%	micro
Chalcidoidea	Tetracampidae	0	14	14	100%	micro
Chalcidoidea	Torymidae	88	1	89	99%	macro
Chalcidoidea	Trichogrammatidae	5	241	246	98%	micro
Chrysidoidea	Bethylidae	4	2	6	67%	macro
Chrysidoidea	Chrysididae	10	0	10	100%	macro
Chrysidoidea	Dryinidae	8	3	11	73%	macro
Cynipoidea	Cynipidae	4	0	4	100%	macro
Cynipoidea	Figitidae	13	57	70	81%	micro
Diaprioidea	Diapriidae	189	105	294	64%	macro
Evanioidea	Gasteruptiidae	1	0	1	100%	macro
Ichneumonoidea	Braconidae	579	212	791	73%	macro
Ichneumonoidea	Ichneumonidae	962	7	969	99%	macro
Mymarommatoidea	Mymarommatidae	1	0	1	100%	macro
Platygastroidea	Platygastridae	5	105	110	95%	micro
Platygastroidea	Scelionidae	11	363	374	97%	micro
Proctotrupoidea	Proctotrupidae	14	0	14	100%	macro
Vespoidea	Formicidae	73	0	73	100%	macro
Vespoidea	Pompilidae	129	0	129	100%	macro
Vespoidea	Sapygidae	4	0	4	100%	macro
Vespoidea	Vespidae	34	0	34	100%	macro
‘Symphyta’		62	0	62	100%	macro

## References

[B9842831] Brooks David R., Bater John E., Clark Suzanne J., Monteith Don T., Andrews Christopher, Corbett Stuart J., Beaumont Deborah A., Chapman Jason W. (2012). Large carabid beetle declines in a United Kingdom monitoring network increases evidence for a widespread loss in insect biodiversity. Journal of Applied Ecology.

[B9842844] Brühl Carsten A., Bakanov Nikita, Köthe Sebastian, Eichler Lisa, Sorg Martin, Hörren Thomas, Mühlethaler Roland, Meinel Gotthard, Lehmann Gerlind U. C. (2021). Direct pesticide exposure of insects in nature conservation areas in Germany. Scientific Reports.

[B9842858] Buffington Matt, Gates Michael (2008). The Fractionator: a simple tool for mining ‘Black Gold’.. Skaphion.

[B9842867] Chimeno Caroline, Hausmann Axel, Schmidt Stefan, Raupach Michael J., Doczkal Dieter, Baranov Viktor, Hübner Jeremy, Höcherl Amelie, Albrecht Rosa, Jaschhof Mathias, Haszprunar Gerhard, Hebert Paul D. N. (2022). Peering into the Darkness: DNA barcoding reveals surprisingly high diversity of unknown species of Diptera (Insecta) in Germany. Insects.

[B9842884] Ekrem Torbjørn, Willassen Endre, Stur Elisabeth (2007). A comprehensive DNA sequence library is essential for identification with DNA barcodes. Molecular Phylogenetics and Evolution.

[B9842907] Elbrecht Vasco, Peinert Bianca, Leese Florian (2017). Sorting things out: Assessing effects of unequal specimen biomass on DNA metabarcoding. Ecology and Evolution.

[B9842916] Elbrecht Vasco, Vamos Ecaterina Edith, Meissner Kristian, Aroviita Jukka, Leese Florian (2017). Assessing strengths and weaknesses of DNA metabarcoding‐based macroinvertebrate identification for routine stream monitoring. Methods in Ecology and Evolution.

[B9842893] Elbrecht Vasco, Bourlat Sarah J., Hörren Thomas, Lindner Angie, Mordente Adriana, Noll Niklas W., Schäffler Livia, Sorg Martin, Zizka Vera M. A. (2021). Pooling size sorted Malaise trap fractions to maximize taxon recovery with metabarcoding. PeerJ.

[B9842926] Engel Michael S., Ceríaco Luis M. P., Daniel Gimo M., Dellapé Pablo M., Löbl Ivan, Marinov Milen, Reis Roberto E., Young Mark T., Dubois Alain, Agarwal Ishan, Lehmann A. Pablo, Alvarado Mabel, Alvarez Nadir, Andreone Franco, Araujo-Vieira Katyuscia, Ascher John S., Baêta Délio, Baldo Diego, Bandeira Suzana A., Barden Phillip, Barrasso Diego A., Bendifallah Leila, Bockmann Flávio A., Böhme Wolfgang, Borkent Art, Brandão Carlos R. F., Busack Stephen D., Bybee Seth M., Channing Alan, Chatzimanolis Stylianos, Christenhusz Maarten J. M., Crisci Jorge V., D’elía Guillermo, Da Costa Luis M., Davis Steven R., Lucena Carlos Alberto S., Deuve Thierry, Fernandes Elizalde Sara, Faivovich Julián, Farooq Harith, Ferguson Adam W., Gippoliti Spartaco, Gonçalves Francisco M. P., Gonzalez Victor H., Greenbaum Eli, Hinojosa-Díaz Ismael A., Ineich Ivan, Jiang Jianping, Kahono Sih, Kury Adriano B., Lucinda Paulo H. F., Lynch John D., Malécot Valéry, Marques Mariana P., Marris John W. M., Mckellar Ryan C., Mendes Luis F., Nihei Silvio S., Nishikawa Kanto, Ohler Annemarie, Orrico Victor G. D., Ota Hidetoshi, Paiva Jorge, Parrinha Diogo, Pauwels Olivier S. G., Pereyra Martín O., Pestana Lueji B., Pinheiro Paulo D. P., Prendini Lorenzo, Prokop Jakub, Rasmussen Claus, Rödel Mark-Oliver, Rodrigues Miguel Trefaut, Rodríguez Sara M., Salatnaya Hearty, Sampaio Íris, Sánchez-García Alba, Shebl Mohamed A., Santos Bruna S., Solórzano-Kraemer Mónica M., Sousa Ana C. A., Stoev Pavel, Teta Pablo, Trape Jean-François, Dos Santos Carmen Van-Dúnem, Vasudevan Karthikeyan, Vink Cor J., Vogel Gernot, Wagner Philipp, Wappler Torsten, Ware Jessica L., Wedmann Sonja, Zacharie Chifundera Kusamba (2021). The taxonomic impediment: a shortage of taxonomists, not the lack of technical approaches. Zoological Journal of the Linnean Society.

[B9843024] Forbes Andrew A., Bagley Robin K., Beer Marc A., Hippee Alaine C., Widmayer Heather A. (2018). Quantifying the unquantifiable: why Hymenoptera, not Coleoptera, is the most speciose animal order. BMC Ecology.

[B9843034] Geiger Matthias F., Moriniere Jerome, Hausmann Axel, Haszprunar Gerhard, Wägele Wolfgang, Hebert Paul D. N., Rulik Björn (2016). Testing the Global Malaise Trap Program - How well does the current barcode reference library identify flying insects in Germany?. Biodiversity Data Journal.

[B9843046] Goulet H., Huber J. T. (1993). Hymenoptera of the world: an identification guide to families.

[B9843054] Hajibabaei Mehrdad, Shokralla Shadi, Zhou Xin, Singer Gregory A. C., Baird Donald J. (2011). Environmental barcoding: a next-generation sequencing approach for biomonitoring applications using river benthos. PLOS One.

[B9843064] Hallmann Caspar A., Sorg Martin, Jongejans Eelke, Siepel Henk, Hofland Nick, Schwan Heinz, Stenmans Werner, Müller Andreas, Sumser Hubert, Hörren Thomas, Goulson Dave, Kroon Hans (2017). More than 75 percent decline over 27 years in total flying insect biomass in protected areas. PLOS One.

[B9843081] Hausmann A., Krogmann L., Peters R., Rduch V., Schmidt S. (2020). GBOL III: Dark Taxa - iBOL Barcode Bulletin. https://ibol.org/barcodebulletin/research/gbol-iii-dark-taxa/.

[B9843414] Karlsson Dave, Hartop Emily, Forshage Mattias, Jaschhof Mathias, Ronquist Fredrik (2020). The Swedish Malaise Trap Project: A 15 Year Retrospective on a Countrywide Insect Inventory. Biodiversity Data Journal.

[B9843099] Lehmann Gerlind U. C., Bakanov Nikita, Behnisch Martin, Bourlat Sarah J., Brühl Carsten A., Eichler Lisa, Fickel Thomas, Geiger Matthias F., Gemeinholzer Birgit, Hörren Thomas, Köthe Sebastian, Lux Alexandra, Meinel Gotthard, Mühlethaler Roland, Poglitsch Hanna, Schäffler Livia, Schlechtriemen Ulrich, Schneider Florian D., Schulte Ralf, Sorg Martin, Sprenger Maximilian, Swenson Stephanie J., Terlau Wiltrud, Turck Angela, Zizka Vera M. A. (2021). Diversity of Insects in Nature protected Areas (DINA): an interdisciplinary German research project. Biodiversity and Conservation.

[B9843129] Lister Bradford C., Garcia Andres (2018). Climate-driven declines in arthropod abundance restructure a rainforest food web. Proceedings of the National Academy of Sciences of the United States of America.

[B9843138] Maes Dirk, van Dyck Hans (2001). Butterfly diversity loss in Flanders (north Belgium): Europe's worst case scenario?. Biological Conservation.

[B9843147] Matthews R. W., Matthews J. R. (1983). Malaise traps: the Townes model catches more insects.

[B9843156] Moser Marina, Ulmer Jonah M., van de Kamp Thomas, Vasilița Cristina, Renninger Maura, Mikó István, Krogmann Lars (2023). Surprising morphological diversity in ceraphronid wasps revealed by a distinctive new species of *Aphanogmus* (Hymenoptera: Ceraphronoidea). European Journal of Taxonomy.

[B9843168] Noyes J. S. (2019). Universal Chalcidoidea Database.. http://www.nhm.ac.uk/chalcidoids.

[B9843176] Piper Alexander M., Batovska Jana, Cogan Noel O. I., Weiss John, Cunningham John Paul, Rodoni Brendan C., Blacket Mark J. (2019). Prospects and challenges of implementing DNA metabarcoding for high-throughput insect surveillance. GigaScience.

[B9843188] Ratnasingham Sujeevan, Hebert Paul D. N. (2007). bold: The Barcode of Life Data System (http://www.barcodinglife.org). Molecular Ecology Notes.

[B9843197] Seibold Sebastian, Gossner Martin M., Simons Nadja K., Blüthgen Nico, Müller Jörg, Ambarlı Didem, Ammer Christian, Bauhus Jürgen, Fischer Markus, Habel Jan C., Linsenmair Karl Eduard, Nauss Thomas, Penone Caterina, Prati Daniel, Schall Peter, Schulze Ernst-Detlef, Vogt Juliane, Wöllauer Stephan, Weisser Wolfgang W. (2019). Arthropod decline in grasslands and forests is associated with landscape-level drivers. Nature.

[B9843221] Shaffer H. Bradley, Fisher Robert N., Davidson Carlos (1998). The role of natural history collections in documenting species declines. Trends in Ecology & Evolution.

[B9843230] Shendure Jay, Ji Hanlee (2008). Next-generation DNA sequencing. Nature Biotechnology.

[B9843239] Srivathsan Amrita, Ang Yuchen, Heraty John M., Hwang Wei Song, Jusoh Wan F. A., Kutty Sujatha Narayanan, Puniamoorthy Jayanthi, Yeo Darren, Roslin Tomas, Meier Rudolf (2023). Convergence of dominance and neglect in flying insect diversity. Nature ecology & evolution.

[B9843254] Ssymank A., Sorg M., Doczkal D., Rulik B., Merkel-Wallner G., Vischer-Leopold M. (2018). Praktische Hinweise und Empfehlungen zur Anwendung von Malaisefallen für Insekten in der Biodiversitätserfassung und im Monitoring. Series Naturalis.

[B9843265] Staab Michael, Gossner Martin M., Simons Nadja K., Achury Rafael, Ambarlı Didem, Bae Soyeon, Schall Peter, Weisser Wolfgang W., Blüthgen Nico (2023). Insect decline in forests depends on species' traits and may be mitigated by management. Communications Biology.

[B9843279] Taberlet Pierre, Coissac Eric, Pompanon François, Brochmann Christian, Willerslev Eske (2012). Towards next-generation biodiversity assessment using DNA metabarcoding. Molecular Ecology.

[B9843289] Townes Henry (1972). A light-weight Malaise trap. Entomological News.

[B9843298] Virgilio Massimiliano, Backeljau Thierry, Nevado Bruno, Meyer Marc (2010). Comparative performances of DNA barcoding across insect orders. BMC Bioinformatics.

[B9843307] Wührl Lorenz, Pylatiuk Christian, Giersch Matthias, Lapp Florian, Rintelen Thomas, Balke Michael, Schmidt Stefan, Cerretti Pierfilippo, Meier Rudolf (2022). DiversityScanner: Robotic handling of small invertebrates with machine learning methods. Molecular Ecology Resources.

[B9843321] Zhang Z. -Q. (2011). Animal biodiversity.

